# Effectiveness of a multi-facetted blended eHealth intervention during intake supporting patients and clinicians in Shared Decision Making: A cluster randomised controlled trial in a specialist mental health outpatient setting

**DOI:** 10.1371/journal.pone.0199795

**Published:** 2018-06-26

**Authors:** Margot Metz, Iman Elfeddali, Marjolein Veerbeek, Edwin de Beurs, Aartjan Beekman, Christina van der Feltz-Cornelis

**Affiliations:** 1 GGz Breburg, Tilburg, The Netherlands; 2 Trimbos Institute, Utrecht, The Netherlands; 3 VU University, EMGO, Amsterdam, The Netherlands; 4 Tilburg University, Tranzo Department, Tilburg, The Netherlands; 5 Foundation for Benchmarking Mental Health Care, Bilthoven, The Netherlands; 6 University of Leiden, Department of Clinical Psychology, Leiden, The Netherlands; 7 GGZ inGeest, Amsterdam, The Netherlands; 8 VU University Medical Centre, Department of Psychiatry, Amsterdam, The Netherlands; TNO, NETHERLANDS

## Abstract

**Objective:**

To investigate the effectiveness of a multi-facetted blended eHealth intervention, called SDM-Digital Intake (SDM-DI), in which patients and clinicians are supported in Shared Decision Making during the intake process.

**Methods:**

The study is a two-arm matched-paired cluster Randomised Controlled Trial in a specialist mental health outpatient setting with two conditions: SDM-DI and Intake As Usual (IAU). Four intake teams were allocated to each arm. All patients who followed an intake, were asked to participate if they were capable to complete questionnaires. Decisional Conflict (DC), referring to patients’ engagement and satisfaction with clinical decisions, was the primary outcome. Secondary outcomes were patient participation, applying Shared Decision Making (SDM), working alliance, treatment adherence and symptom severity. Effects were measured at two weeks (T1) and two months (T2) after intake. Multilevel regression and intention-to-treat analyses were used. Additionally, the influence of subgroups and intervention adherence on DC were explored.

**Results:**

At T1, 200 patients participated (47% intervention, 53% control), and at T2 175 patients (47% intervention, 53% control). At T1 and T2, no differences were found between conditions on DC. Subgroup analyses showed that effects of SDM-DI on DC were not modified by primary diagnoses mood, anxiety and personality disorders. Compared to IAU, at T2, patients reported positive effects of SDM-DI on SDM (β 7.553, p = 0.038, 95%CI:0.403–14.703, *d* = 0.32) and reduction of symptoms (β -7.276, p = 0.0497, 95%CI:-14.544–-0.008, *d* = -0.43). No effects were found on patient participation, working alliance and treatment adherence. Exploratory analyses demonstrated that if SDM was applied well, patients reported less DC (β = -0.457, p = 0.000, 95%CI:-0.518–-0.396, *d* = -1.31), which was associated with better treatment outcomes.

**Conclusion:**

Although, this trial fails to demonstrate that SDM-DI by itself is sufficient to reduce DC, the results are encouraging for further efforts in improving and implementing the SDM intervention.

## Introduction

### Background

Although the beneficial effects of Shared Decision Making (SDM) in mental health care are supported by several studies [1–5], to date there is still much to improve in the degree of patient participation in decision making about treatment [6–8]. Previous research [1–5] pointed out that SDM in mental health care can lead to better informed patients, more patient satisfaction and an improvement in treatment engagement, which, in turn, can have a positive impact on clinical outcomes. Nevertheless, patients in mental health care regularly experience low levels of engagement and satisfaction regarding clinical decision making [8], while they do prefer to be (more) involved in decision making with regard to their treatment [6,7,9–12]. SDM is the collaborative approach in which patients, relatives and clinicians share available information during clinical decision making and where patients, along with relatives, are supported to participate actively in decision making about their treatment [13]. To enhance SDM, it is important to support both patients and clinicians in these relatively new way of working [7,14,15]. Previous studies about the implementation of SDM, have demonstrated the negative influence of the power imbalance between patients and clinicians on the application of SDM in clinical practice. These studies also showed the importance to change both clinicians’ and patients’ attitudes and skills in making shared decisions [7,14].

### Rationale

To stimulate the shift towards SDM from the start of treatment, GGz Breburg, a specialist mental health organisation in the southern part of the Netherlands, invested in the development and implementation of a novel multi-facetted digital SDM intervention in the intake process [16]. This intervention, called SDM-Digital Intake (SDM-DI), consisted of a blended eHealth intervention integrated with the initial Routine Outcome Measurement (ROM), peer workers support and clinicians’ training. ROM implies regular measurements of clinical outcomes during treatment, which provides feedback on the patients’ progress in treatment. The initial ROM is regularly planned in the intake process [17,18]. The SDM-DI initiative aimed to target both sides of the dyad, i.e. patients as well as professionals, in order to foster SDM. The intervention was evaluated in a two-arm cluster Randomised Controlled Trial (RCT). To our knowledge, no previous RCT has investigated such a multi-facetted digital SDM intervention facilitating both patients and clinicians in the intake process.

### Objective and hypotheses

This trial aimed to investigate the effects of SDM-Digital Intake (SDM-DI) on the primary outcome Decisional Conflict (DC), which refers to the degree to which patients were engaged in and felt comfortable about important clinical decisions [8,19], and the secondary outcomes patient participation in decision making, the working alliance, adherence to treatment and symptom severity. It was hypothesized that, compared to the Intake As Usual (IAU), the intervention: 1) diminishes patients’ perception of DC, 2) fosters patients’ participation, 3) stimulates the SDM process, 3) enhances the working alliance between patients and clinicians, 4) leads to more treatment adherence, and 5) improves treatment outcome.

## Materials and methods

### Ethics statement

The Medical Ethics Committee of VU University Medical Centre Amsterdam, The Netherlands reviewed the study and declared that the Medical Research Involving Human Subjects Act (WMO) did not apply to this study (reference number: 2015.434). All participating patients gave written informed consent before filling in the research questionnaires.

### Trial design

This study was a two-arm matched-pair cluster randomised controlled trial. To keep contamination to a minimum, we used a cluster design at team level with pairs of similar teams within the same department, treating a similar population of patients in the same geographical catchment area, which is considered a good procedure in RCTs evaluating interventions at clinicians’ level [20]. In this study, the application of SDM-Digital Intake (SDM-DI) was compared with the Intake As Usual (IAU). The design of this study has been described in more detail elsewhere [16]. The trial is registered in the Dutch trial register with number: NTR5677, registration date: 17 January 2016 (http://www.trialregister.nl/trialreg/admin/rctview.asp?TC=5677). Results are presented in accordance with the CONSORT statement for cluster randomised controlled trials [21].

### Settings and location

This trial was conducted in four outpatient departments of the specialist mental health care organisation GGz Breburg located in the southern part of the Netherlands. The SDM-DI intervention was intended to be suitable for patients with various diagnoses (depression, anxiety and personality disorders), and was therefore tested in two outpatient departments specialised in depression and anxiety disorders, and two outpatient departments specialised in personality disorders. Each of the two departments, which were specialised in the same patient group, where working in separate catchment areas. The four participating departments consisted of two multidisciplinary intake-teams each, in which initial treatment decisions are made.

### Participants and eligibility criteria

In total eight intake teams from four departments operating in two catchment areas participated in this trial. Research assistants consecutively invited each new patient, for whom a full intake (SDM-DI or IAU) was planned, to participate in this study. Patients were excluded if they did not speak or read the Dutch language or were incapable to complete questionnaires because of cognitive functioning or an ongoing crisis. Patients enrolled after receiving face to face and written information about the study given by the research assistants, and after giving written informed consent.

### Randomisation of clusters

Cluster randomisation at the level of the four pairs of intake teams was performed using computer-generated random numbers. According to the matched-pair cluster randomised design, the randomisation sequence was performed per block of two intake-teams using a SPSS-syntax which generated a random number (0 = control/IAU, 1 = intervention/SDM-DI). This syntax was prepared by a data manager with no involvement in this trial. The random allocation sequence was conducted by the co-author IE, prior to the start of the intervention and data collection of this study, independent of the participating teams and the primary researcher MM, who coordinated and conducted the trial.

Four teams were randomised to the intervention group and four teams to the control group. To reduce the risk of contamination from the intervention to the control condition, the participating teams had their own multidisciplinary team consultation. Furthermore, clinicians and patients participated in one condition. Patients who were planned for an intake consultation with clinicians of the intervention group, were automatically assigned to the intervention and received SDM-Digital Intake (SDM-DI). Patients who had the first intake appointment with clinicians of the control group, followed the intake as usual (IAU). Planning of these intake consultations was conducted by secretaries with no involvement in the study, according to the availability of time in the agendas of patients and intake clinicians. There was no influence of other factors on this planning process.

### Blinding

Due to cluster randomisation at team level and the nature of the intervention (i.e. clinicians had to guide the digital intake approach and patients did follow eHealth modules), blinding of the clinicians and patients was not feasible. To reduce the risk of bias, research assistants, independent of the research team and participating teams, performed the inclusion of patients and carried out the data collection. During the inclusion process, the independent research assistants were blinded to the allocation of the condition.

### Interventions

The multi-facetted intervention, called SDM-Digital Intake (SDM-DI), aimed to target both sides of the dyad, i.e. patients as well as professionals, in order to foster SDM. SDM-DI is formed by a digital intake approach incorporating a blended eHealth intervention integrated with Routine Outcome Monitoring (ROM), support of peer workers and training of clinicians. Patients participating in the intervention group were also stimulated to involve a relative in their intake process. The intervention is briefly described below. A comprehensive description can be found elsewhere [16].

#### Blended eHealth intervention integrated with ROM

Two eHealth modules were offered in order to explore treatment needs, expectations and preferences of patients aiming to support patients in preparing themselves, along with relatives, for the intake consultations. The initial ROM, which measured symptom severity, was integral part of the eHealth modules. ROM implies regular measurements of clinical outcomes during treatment, which provides feedback on the patients’ progress in treatment. The initial ROM is regularly planned in the intake process [17,18]. While following eHealth, patients completed the first ROM, had direct access to their ROM results, and got the opportunity to have contact with trained peer workers. Peer workers have life experience in mental illness and treatment, and could help patients while following the eHealth modules and with preparations for the intake and choices in treatment.

#### Intake consultations

The results of the completed modules and ROM were visible for patients and clinicians as well. Patients were stimulated to use their own feedback reports, with personalised graphics about their mental health problems and the impact on daily life, to prepare the intake consultations and bring them to these consultations. The assertion was that patients who were better prepared for the intake, would be more able to actively participate in the dialogue with their intake clinicians about choices in treatment. To make sure that patients and clinicians have sufficient time to discuss the preparation by eHealth and ROM, in the intervention group, the first intake consultation was extended from 60 to 90 minutes.

#### Training of clinicians

The clinicians of the intervention group followed a training of three half-day sessions aimed to stimulate and facilitate the new way of working. The training was given by a trainers couple formed by a peer worker and clinician and contained explanations of, instructions on and exercises in recovery supported care and the application of SDM [22] using eHealth and ROM as sources of personalised information. During the course of the study, the intervention teams organised at least one supervision session, where clinicians evaluated and discussed their experiences in SDM-DI with colleagues.

#### Intake as usual

The control group consisted of an Intake As Usual (IAU), which means that patients in the control group did not follow the blended eHealth intervention, did not complete the initial ROM in the eHealth portal, had no digital access to their own ROM results and could not consult a peer worker in the intake process. The time of the first intake consultation in the control group was standard 60 minutes. Furthermore, the intake clinicians of the control group did not follow training in recovery supported care and the application of SDM using eHealth and ROM. In both arms, the multidisciplinary team consultation fulfilled the usual role of checking the quality of the treatment choices.

### Outcomes

#### Measurements

To prevent socially desirable answers and an undesired influence of the research team or clinicians on the results several precautions were taken. First, data were collected by independent research assistants. Second, the outcomes, Decisional Conflict (DC), patient participation, SDM process and working alliance, were measured with separate instruments that were included for research purposes only. Third, the results on these research instruments were not visible for patients and clinicians during intake and treatment. The research instruments were completed two weeks (T1) and two months (T2) after intake, and thus measured both the effects of the intervention. Furthermore, two weeks after intake (T1), clinicians answered questions about SDM regarding their patients. Patients and clinicians received a link by email to complete the questionnaires and if necessary received, after 7, 9 and 14 days, reminders by email or phone. If patients did not use internet, they received paper questionnaires by post.

The other outcomes no-show, drop-out and symptom severity (i.e. ROM) were derived from the electronic patient records. Participants in the intervention and control group completed the regular initial ROM measurement at baseline (T0) and the follow up ROM measurements at T1 and T2.

#### Primary outcome

Decisional Conflict (DC) was measured in patients using the revised, validated Decisional Conflict Scale (DCS) [19], which was translated into Dutch [22]. Each of the 16 items is scored from 0 (strongly agree) to 4 (strongly disagree). Besides a total score, the DCS includes five dimensions (information, support, clarification of values, certainty and decision quality). Higher scores indicate more DC, which means that patients report to have achieved less information, less support, less clarification, less certainty and poorer decision quality about decision making. To calculate the total scale and scores of the five dimensions the item scores were summed, divided by the number of items and multiplied by 25. The scores thus range from 0 to 100 [19]. The internal consistencies of the total score and five dimensions of the Dutch version of the DCS calculated in this study population were good (Total scale α = .95, subdomains α ≥ .77). To compare patients’ and clinicians’ views on DC, additionally DC was measured on a Visual Analogue (VAS) 10-point scale (item: ‘to what extent do you agree with the decision taken?’), filled out at T1 by both patients and clinicians (regarding their patients).

#### Secondary outcomes

We used the following validated self-report questionnaires to measure the secondary outcomes: Patient Participation Questionnaire (PPQ) [16], the patients’ and clinicians’ versions of the Shared Decision Making-Questionnaire-9 (SDM-Q-9) [23], Patient-Doctor Relationship Questionnaire-9 (working alliance) [24] and Symptom Questionnaire-48 (SQ-48) [25,26]. The Cronbach’s alphas of the total scores of these secondary outcome questionnaires in this study population were good (PPQ α = .90; SDM-Q-9 patient α = .95; SDM-Q-9 clinician α = .86; PDRQ-9 α = .97; SQ-48 α = .93). At the end of the study, patients received an additional question about the extent to which they achieved their personal treatment goals [16]. Finally, treatment adherence was assessed by the number of missed appointments (no-shows) and patients who did not want to proceed with treatment (treatment drop out) [16].

#### Intervention integrity

To check the intervention integrity, process variables were collected. These variables report the degree of completion of eHealth modules and the frequency of consulting peers.

#### Patients’ and clinical characteristics

Patients’ and clinical characteristics were collected at baseline. The following data were registered in the Electronical Patient Records (EPR): gender, age, educational level, primary diagnosis, symptom severity, motivation to start treatment (own initiative or by pressure of the social environment), length of previous treatment (at GGz Breburg in weeks) and length of waiting time (from registration to first intake consultation in weeks).

### Sample size

A sample size calculation prior to the study [16] was performed to detect a difference between the two arms with an expected clinically relevant medium effect size on the primary outcome patients’ rated Decisional Conflict. We were, as far as we know, the first to examine the effects of such an intensive multi-facetted eHealth intervention during intake supporting patients and clinicians in Shared Decision Making, performed in specialist mental health care using Decisional Conflict experienced by patients as a primary outcome. Therefore, it was difficult to determine an exact estimate of the effect size for the primary outcome. We used a medium effect size of d = 0.5, because this is considered to be a clinically meaningful effect.

A sample size of 65 patients per arm was needed to obtain an usual power of β = .80 with an alpha set at 0.05 (two tailed) [27]. Adjustment for clustering within teams assumed an expected intra cluster correlation coefficient (ICC) of 0.01. Although the cluster variation can rarely be estimated in advance, we expected the ICC at team level to be low because of the stratified randomisation between clusters (matched-paired design) of participating teams from a single mental health organisation [28,29]. Moreover, a reanalysis of cluster-based studies in primary care [28] demonstrated also a low level of clustering (median ICC of 0.01), even between different general practices.

We calculated [21,30] that with an ICC of 0.01 and an inflation factor of 1.18 at team level a sample size of 77 completers per arm was needed (Design Effect (DE) = 1 +(m-1) * ICC (m = number of subjects in a cluster) = > DE = 1 + (77/4-1)*0.01 = DE 1.18). Taking into account a dropout rate of 10%, we calculated that at least 88 patients had to be included per arm to certify sufficient power.

### Statistical methods

The analyses were conducted using intention-to-treat principles. Multi-level analyses were performed in MLwiN 2.21 software. The other analyses were performed in SPSS 19.0.

#### Descriptives and drop-out analyses

Descriptive analyses were conducted in order to analyse patients’ characteristics and intervention integrity. Attrition analyses were performed by means of a logistic regression analysis in order to test for selective drop-out between T1 and T2. If participants had answered at least 80% of the items on a questionnaire, missing items were imputed with the mean value of the completed items.

#### Primary and secondary outcomes

To correct for possible clustering of the data and to handle missing data [31], the analyses of the primary and secondary outcomes, which measured effects at T1 and T2, were performed using linear multilevel regression analysis (MLA) with three levels (teams, couples of intake-clinicians and patients). Severity of symptoms, measured by SQ-48 at three measurement moments (T0, T1, T2) were analysed using longitudinal MLA. We performed this longitudinal analysis with four levels (teams, couples of intake-clinicians, patients and multiple measurements within patients) and adjusted for the baseline score T0. Both the overall effects (all measurements T1 and T2) as the effects at the separate measurement moments (T1, T2 separately) were calculated. The analyses of the outcomes were performed with a two-tailed 0.05 significance level. The effect sizes (Cohen’s *d*) were calculated by dividing the between-group difference by the pooled SD. The thresholds for interpreting the effect size were: Small 0.00–0.32, Medium 0.33–0.55 and Large ≥ 0.56 [32].

#### Additional analyses

The differences in patients’ and clinicians’ views on the application of Shared Decision Making (SDM-Q-9) and agreement with clinical decisions (DC VAS) were tested using dependent t-tests. Furthermore, post-hoc analyses were performed for the primary outcome Decisional Conflict (DC). We assessed potential effect modification by primary diagnoses, motivation for treatment, previous treatment and waiting time. Interactions were tested for significance at a p-value of 0.1. We also investigated the influence of intervention integrity on DC. Finally, we explored the influence of applying SDM on the primary outcome DC and the association between DC and symptom severity.

## Results

### Recruitment and participants flow

**Fig 1:**
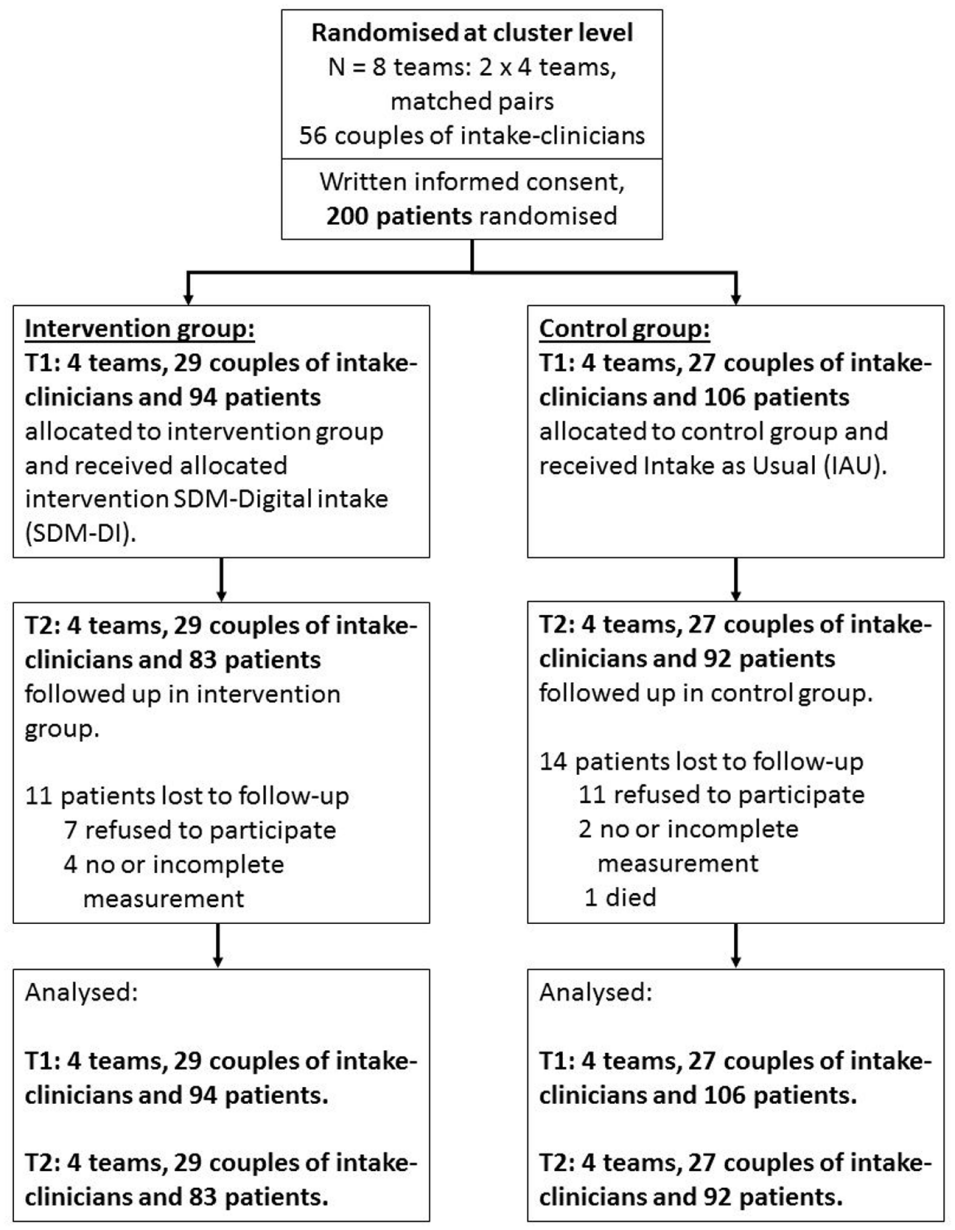
Flow chart RCT Shared Decision Making in a digital intake approach (Consort 2010).

Inclusion started in October 2016 and ended in June 2017, when the required sample size was reached. Follow-up measurements were completed in August 2017. Eight teams (four intervention teams with in total 29 couples of intake clinicians, who jointly performed the intake, and four control teams with in total 27 couples of intake clinicians) of four departments from one specialist mental health care organisation participated in the trial. As shown in Fig 1, 200 patients (94 intervention, 47%; and 106 control, 53%) gave written informed consent and responded to the first measurement (T1). These 200 patients were included in the analyses. In total 175 patients (83 intervention, 47%; and 92 control, 53%) filled out the follow-up measurement at 2 months (T2). The range of participating patients per team was 17 to 37 (mean 25 patients) and per couple of intake clinicians the range was 1 to 23 patients (mean 4.4 patients).

### Loss to follow-up

The dropout rate of patients between T1 and T2 was 13% (11 intervention, 14 control). Numbers of dropout were not significantly different between the conditions and dropout rates were not significantly associated with any patient characteristics (gender, age, educational level, primary diagnosis, treatment motivation). Reasons for dropout were: 1) no longer willing to fill out research questionnaires (i.e. withdrawal of informed consent) (80%), 2) not responding to reminders (17%), 3) death during study period (3%).

### Baseline characteristics

Table 1 presents patients’ and clinical characteristics at baseline in terms of gender, age, educational level, primary diagnosis, symptom severity (SQ48 total score) at baseline, treatment motivation, length of waiting till first intake consultation (in weeks) and previous treatment at GGz Breburg (in weeks). At baseline, these characteristics were equally distributed over the two arms.

**Table 1:** Patients’ characteristics.

		Total study population (n = 200)	Intervention group (n = 94)	Control group (n = 106)
**Gender**	**Female**	132 (66%)	66 (70,2%)	66 (62,3%)
	**Male**	68 (34%)	28 (29,8%)	40 (37,7%)
**Age**	**Mean age (sd)**	38,3 (10,2)	38,6 (10,6)	38,0 (9,8)
**Educational level**	**Primary school or Lower secondary education (low)**	49 (24,5%)	20 (21,3%)	29 (27,4%)
	**Higher secondary or intermediate vocational education (middle)**	115 (57,5%)	56 (59,6%)	59 (55,7%)
	**Higher vocational education or university/Bachelor’s or Master’s degree (high)**	36 (18,0%)	18 (19,1%)	18 (17,0%)
**Primary diagnosis** *	**Personality disorder**	101 (51,8%)	44 (48,9%)	57 (54,3%)
	**Anxiety disorder**	40 (20,5%)	20 (22,2%)	20 (19,0%)
	**Mood disorder**	39 (20,0%)	21 (23,3%)	18 (17,1%)
	**Other disorders** **	15 (7,7%)	5 (5,6%)	10 (9,5%)
**SQ48 total score (symptom severity, at T0)**		73,3 (23,1)	71,4 (23,0)	75,7 (23,2)
**Treatment motivation** ***	**Own initiative**	149 (74,9%)	63 (67,7%)	86 (81,1%)
	**Influence of others (social environment)**	50 (25,1%)	30 (32,3%)	20 (18,9%)
**Length of waiting (weeks)**		15,7 (55,0)	13,4 (33,4)	17,7 (68,9)
**Previous treatment (weeks)**		74,9 (120,5)	74,5 (110,2)	75,2 (129,4)

*Missing variable/primary diagnosis not registered in EPR n = 5

**Other disorders: Group with a diversity of other disorders i.e. Autistic disorder, Attention deficit hyperactivity disorder, Pedophilia, Impulse control disorder, Adaptive disorder, Partner-relationship problem, Substance dependency, Psychotic disorder NAO, Undifferentiated somatoform disorder, Hypochondria, Disorder in the body experience.

***Missing variable/treatment motivation not registered in EPR n = 1

### Intention to treat analyses

Tables 2 and 3 show the effects of the intervention SDM-DI on the primary and secondary outcomes. At first, overall effects (T1 and T2) were reported. Second, the results at T1 and T2 were shown separately.

**Table 2:** Effects of SDM-DI on the primary outcome.

Primary outcome Decisional Conflict															
	Overall effect (T1 and T2)					T1					T2				
	mean T1 and T2 (sd)	β	95%CI	p	Effect size	mean T1 (sd)	β	95%CI	p	Effect size	mean T2 (sd)	β	95%CI	p	Effect size
**Total**	I 42.78 (sd 16.70)	-1.835	-7.237–3.567	0.506	-0.10	I 42.97 (18.45)	-3.565	-10.000–2.870	0.278	-0.10	I 41.33 (19.37)	0.162	-6.545–6.869	0.964	0.01
	C 42.93 (15.97)					C 45.97 (19.59)					C 42.57 (19.21)				
**Information**	I 47.49 (20.76)	0.508	-6.603–7.619	0.889	0.02	I 48.48 (23.76)	-0.858	-9.092–7.376	0.838	-0.04	I 45.88 (22.64)	2.111	-6.433–0.655	0.628	0.09
	C 45.74 (17.85)					C 49.10 (23.45)					C 42.98 (21.97)				
**Support**	I 38.52 (16.95)	-2.119	-7.942–3.704	0.476	-0.10	I 38.16 (20.83)	-3.195	-10.198–3.808	0.371	-0.15	I 38.89 (19.41)	-0.884	-8.187–6.419	0.813	-0.04
	C 38.37 (18.29)					C 40.28 (22.50)					C 38.11 (21.13)				
**Clarification**	I 45.35 (19.21)	-3.044	-9.765–3.677	0.375	-0.13	I 46.88 (23.27)	-5.930	-13.907–2.047	0.145	-0.24	I 43.52 (23.46)	0.289	-8.025–8.603	0.946	0.01
	C 45.88 (19.19)					C 51.72 (25.21)					C 41.57 (22.70)				
**Certainty**	I 46.47 (20.48)	-2.346	-7.307–2.615	0.354	-0.10	I 45.17 (22.38)	-5.892	-12.701–0.917	0.090	-0.26	I 47.53 (24.95)	1.651	-5.536–8.838	0.652	0.06
	C 47.77 (20.37)					C 51.06 (22.85)					C 45.88 (25.93)				
**Quality**	I 37.74 (17.32)	-1.236	-5.474–3.002	0.567	-0.06	I 37.86 (18.46)	-1.912	-7.749–3.925	0.521	-0.10	I 38.43 (22.06)	-0.479	-6.639–5.681	0.879	-0.02
	C 38.41 (17.22)					C 39.77 (21.43)					C 38.90 (20.18)				
**VAS DC patient**	I 6.47 (2.63)	-0.048	-0.993–0.897	0.921	-0.01	I 5.55 (3.51)	-0.294	-1.223–0.635	0.535	-0.08	I 6.75 (3.12)	0.505	-0.393–1.403	0.270	0.16
	C 6.24 (2.94)					C 5.84 (3.56)					C 6.24 (3.20)				
**VAS DC clinician**	Not applicable, from clinician’s perspective only T1 available.					I 8.80 (1.94)	0.083	-0.105–0.271	0.387	0.17	Not applicable, from clinician’s perspective only T1 available.				
						C 8.17 (2.30)									

**Table 3:** Effects of SDM-DI on the secondary outcomes.

**Secondary outcomes Patient Participation (PPQ), Shared Decision Making (SDM-Q-9), Working Alliance (PDRQ-9), Symptom severity (SQ-48)**															
	**Overall effect (T1 and T2)**					**T1**					**T2**				
	**Mean T1 and T2 (sd)**	**β**	**95%CI**	**p**	**Effect size**	**mean T1 (sd)**	**β**	**95%CI**	**p**	**Effect size**	**mean T2 (sd)**	**β**	**95%CI**	**p**	**Effect size**
**PPQ**	I 32.88 (8.53)	0.122	-2.734–2.978	0.933	0.01	I 30.31 (9.51)	-0.302	-3.650–3.046	0.860	-0.03	I 34.53 (10.82)	0.100	-2.944–3.144	0.950	0.01
	C 32.93 (8.48)					C 30.42 (10.53)					C 34.43 (10.01)				
**SDM-Q-9 patient**	I 61.83 (20.57)	6.609	-0.874–14.092	0.083	0.26	I 53.88 (28.20)	6.443	-0.991–13.877	0.089	0.25	I 62.47 (22.13)	7.553	0.403–14.703	0.038	0.32
	C 55.34 (22.79)					C 60.32 (24.12)					C 54.91 (25.65)				
**SDM-Q-9 clinician**	Not applicable, from clinician’s perspective only T1 available.					I 69.08 (17.20)	2.203	-3.238–7.644	0.427	0.15	Not applicable, from clinician’s perspective only T1 available.				
						C 65.67 (12.34)									
**PDRQ-9**	I 3.30 (.97)	0.159	-0.217–0.535	0.408	0.13	I 3.15 (1.15)	0.154	-0.297–0.605	0.503	0.12	I 3.32 (1.11)	0.089	-0.258–0.436	0.615	0.08
	C 3.18 (1.05)					C 3.02 (1.32)					C 3.23 (1.23)				
	**Overall effect (T1 and T2)**					**T1** (median: 33 days after ROM T0)					**T2** (median: 60 days after ROM T0)				
		**Β**	**95%CI**	**p**	**d**	**mean T1 (sd)**	**β**	**95%CI**	**p**	**d**	**mean T2 (sd)**	**β**	**95%CI**	**p**	**d**
**SQ-48**	I 69.31 (17.13)	-4.975	-12.217–2.267	0.178	-0.24	I 69.80 (21.98)	-3.920	-10.549–2.709	0.247	-0.17	I 64.18 (15.27)	-7.276	-14.544–-0.008	0.0497	-0.43
	C 71.61 (20.09)					C 75.05 (24.01)					C 67.60 (18.45)				

C, Control Group; I, Intervention Group; CI, Confidence Interval.

#### Primary outcome

In the intention-to-treat analyses, no significant differences between SDM-DI and IAU on the total scale and subdomains of the primary outcome DC were found (Table 2). We found no evidence for clustering of effects on the primary outcome at team level (ICC = 0), which was the unit of randomisation. At the level of intake clinicians we found a significant cluster effect with an ICC of 0.10. No significant differences were found between the arms for the patients’ and clinicians’ reported version of the DC VAS scale. When comparing the patients’ and clinicians’ reported DC VAS at T1, irrespective of the condition, clinicians scored more positive about the application of SDM (mean_diff_ 2.746, sd 3.873, p<0.001, 95%CI 2.234 to 3.258).

#### Secondary outcomes

The overall effect (T1 and T2) and the difference at T1 on the secondary outcome (Table 3) the degree of applying SDM according to patients (SDM-Q-9 patient total scale) were not significant between the two arms. However, at T2, significant differences were found between the two arms in favour of the intervention group with regard to the application of SDM (β 7.553, p<0.05, 95%CI: 0.403 to 14.703). The difference between arms at T2 showed an effect size of 0.32.

Looking at the clinicians’ reported SDM-Q-9, completed at T1, the results did not differ significantly between the two conditions. When comparing the patients’ and clinicians’ reported SDM-Q-9 at T1, irrespective of the condition, clinicians scored more positive about the application of SDM (mean_diff_ 11.456, sd 28.739, p<0.001, 95%CI 7.310 to 15.602).

Regarding severity of symptoms (SQ-48 total scale), the overall effect (T1 and T2) and the difference at T1 were not significant between the two arms (Table 3). However, at T2, the intervention group scored significant better (β -7.276, p<0.05, 95%CI: -14.544 to -0.008). This measurement was completed 60 days (median) after baseline. Thus, after a period with a median of 60 days, patients in the intervention group reported significantly less symptom severity compared to the control group. The effect size was medium (*d* = -0.43).

Finally, we found no significant effects of SDM-DI on the secondary outcomes (Table 3): working alliance and patient participation. Also, adherence to treatment (drop out and no-shows) and achievement of treatment goals (β 0.059, p = 0.364, 95%CI -0.068 to 0.186, *d* = 0.13) did not differ between the two arms. In the intervention group the treatment drop out percentage was 4.3% (4 patients) and in the control group 2.8% (3 patients). The mean number of no-shows of treatment consultations was in each arm the same (mean 0.02 per patient).

#### Ancillary analyses

Subgroup analyses (Table 4) for the primary outcome patient reported Decisional Conflict (DC) showed no significant interaction effects between the primary diagnoses mood, anxiety and personality disorders and trial condition, which means that the effect of SDM-DI on DC was not influenced by these primary diagnoses for which patient groups the intervention was intended. We also found no significant interaction effects on the primary outcome of the parameters treatment motivation (p = 0.114), treatment history (p = 1.000) and waiting time (p = 0.475) with trial condition.

**Table 4:** Overall results (T1 and T2) of SDM-DI on the primary outcome DC for diagnosis groups.

		Decisional Conflict Total score					
	Diagnosis groups	mean T1 (sd)	mean T2 (sd)	β	95% CI	p-value	Effect size
**Personality disorder**	I 44 patients	I 46.21 (20.60)	I 42.69 (20.64)	-1.582	-8.722–5.558	0.664	-0.12
	C 57 patients	C 46.76 (15.83)	C 44.09 (18.63)				
**Anxiety disorder**	I 20 patients	I 38.91 (15.28)	I 41.67 (23.05)	-2.798	-12.181–6.585	0.559	-0.14
	C 20 patients	C 44.41 (23.65)	C 40.89 (16.98)				
**Mood disorder**	I 21 patients	I 40.23 (18.56)	I 41.53 (14.01)	-2.221	-12.101–7.659	0.660	-0.12
	C 18 patients	C 42.10 (21.77)	C 44.17 (21.62)				
**Other disorders**	I 5 patients	I 47.66 (15.44)	I 55.08 (10.32)	15.142	-0.765–31.049	0.062	0.84
	C 10 patients	C 45.78 (21.96)	C 25.16 (17.81)				

Exploratory analyses revealed that, irrespective of the condition, a better application of SDM, according to the patient reported SDM-Q-9, led to significantly less DC (β = -0.457, p<0.001, 95%CI: -0.518 to -0.396) with a large effect size (*d* = -1.31). We also explored the influence of DC on treatment outcomes. We found significant positive associations between less DC at T2 and better clinical outcomes at T2 (β = 0.227, p<0.05, 95%CI:0.002 to 0.452). This effect size was medium (*d* = 0.45). Although, 77% of the patients in the intervention group completed the first eHealth module and 39% the second module, we found no association between the degree of completion of the eHealth modules and the level of patient reported DC (p = 1.000). Finally, peer workers were hardly consulted. Therefore, we could not explore the association between the frequency of consulting peer workers and the primary outcome of this trial.

No adverse or unintended effects of the SDM-DI intervention or IAU were reported.

## Discussion

### Main findings

This study presents the results of a cluster randomised controlled trial on the effects of SDM-Digital Intake (SDM-DI) in specialist mental health care aimed to foster Shared Decision Making. Compared to the Intake As Usual (IAU), no significant effect of SDM-DI was found on the primary outcome Decisional Conflict (DC) reported by patients and also no significant influence was shown on the DC VAS scale reported by patients and clinicians. However, at T2 compared to the control group, patients in the intervention arm reported a significantly better application of SDM (*d* = 0.32). At T1, the differences in the SDM-Q-9 scores, reported by patients and clinicians, between the two arms were not significant. Looking at treatment outcome, measured by the SQ-48, we found a significant positive intervention effect at T2. Patients of the intervention group, reported more symptom reduction at T2, with a medium effect size (*d* = -0.43). The other secondary outcome parameters, patient participation, working alliance and treatment adherence did not differ between the two arms. Irrespective of the condition, a better application of SDM according to patients was associated with less DC (*d* = -1.31), which in turn had a positive influence on treatment outcome (*d* = 0.45).

### Strengths and limitations

First strength of the study design is the matched pair randomisation of similar intake teams, which reduced the risk of confounding shown by the balance in patients’ characteristics in the two arms. Second, because independent research assistants, who were blinded for the study arm during inclusion, asked all patients who met the inclusion criteria to participate in the trial, selection bias was prevented as much as possible. Third, the mainly independent data collection, coordinated by independent research assistants, with separate research instruments apart from the intake intervention, which results were not visible at patient level during the intake and treatment, prevented socially desirable answers of patients and undesired influence of the research team or clinicians on the results. Only the regular ROM, measuring symptom severity, was used by both arms during intake and treatment. Furthermore, the external validity of this study to the patient population with primary diagnoses mood, anxiety or personality disorders treated in Dutch specialist mental health care proved to be good. Finally, we used multilevel analyses to correct for the clustering of the results at the levels of teams and intake-clinicians. At the team level, which was our level of randomisation, we did not find variability between clusters, probably because of the matched pair cluster randomisation between two equal teams, which belong to the same mental health organisation. At the level of intake clinicians we found a significant cluster effect with an ICC of 0.10. This finding can be explained by the nature of the intervention and the association between the level of SDM with the collaboration style, attitude and skills of the clinicians. Previous research among patients in mental health care showed the association of the patient-clinician working alliance on patient participation in decision making [33–35]. Patients even indicate that the quality of the relationship with their clinician is the most important component that influences SDM [33, 34]. In fact, this is confirmed in our study by the clustering of results at clinicians’ level. In addition, training of the clinicians was an important part of the intervention. A high clustering of the effects at clinicians’ level, can probably also be explained by the fact that the training of clinicians has not the same impact on the participants. The effect of the training might be dependent on the initial level of SDM application and the learning attitude of the clinicians. Subsequently, we recommend to get more insight in the association of clinicians’ characteristics on the application of SDM. If we have more insight in these associations, we were able to tailor future interventions to specific characteristics and needs of clinicians.

This study also has a number of limitations that could have influenced the results. Although, we adopted a cluster randomised controlled design to reduce the risk of cross over effects between the two arms, contamination between the clinicians of the teams cannot be ruled out completely and may have weakened the effects found. Cross over effects between patients were unlikely, because they followed the intake and treatment individually and hence did not meet and know each other. Furthermore, because patients and clinicians were not blinded for the design, it was possible that patients and clinicians from the control teams, made additional efforts in SDM. Research assistants were partially blinded for the study arm, however they could have hardly influenced the results, because the outcome parameters were measured by self-report questionnaires, completed by patients and clinicians. As described more comprehensively in the section ‘interpretation and clinical implications’, the uptake of the intervention was not optimal, especially not on the patients’ side of the dyad. Finally, to discuss the results of the intake module, in the intervention group the initial intake consult took 30 minutes longer compared to the control group. Although, previous research did not show that applying SDM takes more time [36], the extra time of the first intake consultation in the intervention group could be a confounder, because it might have given more possibilities for the patient and clinician to go through the steps in the SDM process carefully. Maybe, therefore patients in the intervention group may have felt more support to participate actively in decision making [7].

### Generalizability

This study was performed in real world clinical practice. Departments of two catchment areas (urban, semi-urban and rural as well) participated in this trial. The participating departments were specialised in the treatment of patients with mood, anxiety and personality disorders. Except of patients who did not speak and read the Dutch language or were unable to answer questionnaires, because of an ongoing crisis or cognitive functioning, all patients were asked to participate. Moreover, the population of 200 participating patients, which was in balance between the two arms, was larger than the estimated sample size of 176. Taken together, it is reasonable to assume that the study results are generalizable to the patient population with mood, anxiety and personality disorders treated in Dutch specialist mental health care. Regarding the exclusion criteria and the focus of the intervention on three diagnosis groups, the current study might not be generalizable to a broader patient population with other diagnoses and patients who were incapable to answer questionnaires.

### Interpretation and clinical implications

To our knowledge, no previous RCT has investigated a multi-facetted intervention aimed to improve SDM in specialist mental health care, targeting both patients’ and clinicians’ behaviour during the intake process. Although previous research [7,14,15] pointed out that to enhance SDM it is important to support both sides of the dyad, until now it was unclear whether patients benefit from the implementation of such a novel combined SDM, blended eHealth, ROM and peer support initiative facilitating both patients and clinicians during the intake process in routine clinical practice. In contrast with our hypotheses we found no significant differences between SDM-DI and IAU regarding Decisional Conflict (DC) experienced by patients, which was our primary outcome. We also did not find effects on the secondary outcomes patient participation, working alliance and treatment adherence. However, in concordance with our expectations, in the intervention arm patients reported that the application of SDM by clinicians was better, and they also reported less symptom severity. Moreover, it was very encouraging to see that a higher level of applying SDM led to less DC, which was positively associated with reduction of symptoms.

The lack of effects in the intervention arm on the primary outcome and some of the secondary outcome parameters, might be explained by the fact that the uptake of SDM-DI in the intervention arm was not sufficient. Possibly, regarding the less than optimal intervention integrity and no significant effect on the secondary outcome patient participation, more support was needed on the patients’ side of the dyad to foster a higher level of patient participation in decision making, which in turn might reduce DC. Whereas, due to the training in SDM skills, clinicians participating in the intervention group actually applied the SDM-steps during intake consultations. Hence, compared to the control group, patients in the intervention group reported a higher level of SDM applicated by clinicians, while this is probably not enough to influence patient reported DC. Nevertheless, patients in the intervention group reported less symptom severity, while we hypothesized this improvement in treatment outcome as a result of reduced DC. In line with this expectation, explorative analyses showed the association between less DC and less symptom severity. A possible explanation of the positive findings on symptom severity in the intervention arm without a change in DC might be that the personalised, recovery oriented attention which was applied in the intervention group, could also influence treatment outcome directly. Looking at the level of DC among patients (Table 2), we can conclude, that there is still room for improvement, because patients in specialist mental health care experience relatively high levels of DC [8], which also applies to patients participating in this study.

To improve the uptake of the intervention, more time and efforts are needed to change the attitudes of clinicians and patients more fundamentally towards SDM [7,11,37,38], in this study especially on the patients’ side of the dyad. Lessons from the implementation of SDM pointed out that the implementation of SDM is a challenging process, which need a bundle of interventions targeting to foster cultural change among patients and clinicians towards SDM as usual practice [11,38,39]. Furthermore, previous research about the implementation of eHealth demonstrated the importance of organising sufficient support to clinicians (i.e. regular supervision sessions) targeting to integrate the application of eHealth tools in their daily working practice [40]. To prepare patients for their active role and give them greater confidence in decisional involvement, patients might need more flexible, personalized support, training and better introduced (digital) tools [7,15,37,41,42]. Previous research and feedback from patients and clinicians, who participated in the intervention group of this study, showed that the implementation of SDM in the intake could have been improved if the eHealth modules with ROM had been better introduced to patients, for example before the intake consultation by peer workers, making it more clear to patients how these tools could help them during the intake process [43,44]. In addition, feedback from the participants showed that if the design of the eHealth modules was more simplified, it would be more attractive to follow the eHealth modules completely.

At the same time, we have limited insight in what happened with patients while following the eHealth modules and we also know less about patients’ preferences about using eHealth and peer support. We only have insight in the percentage technically completed eHealth modules and the fact that peer workers were hardly contacted in the intake process. In future research, it would be interesting to explore these user patterns and preferences, and also investigate the associations between the use of eHealth and peer support with patients characteristics, the level of participation in decision making and treatment outcome. This could give us more insight in which way of working with eHealth and peer support works for whom.

Although, this is a negative trial and we found limited effects of SDM-DI on the secondary outcomes, it was encouraging to find that, irrespective of the condition, a better application of SDM, leads to less DC experienced by patients, which in turn was associated with more symptom reduction. This finding was in line with previous, national research to shared decision making using ROM in Dutch specialist mental health care, where the positive influence of a higher level of SDM on reduced DC, and the effects on treatment outcomes were also shown [22].

### Conclusions

This innovative study stimulated SDM at both sides of the patient-clinician dyad using a combination of digital and face-to-face methods, i.e. digital exploration of patients’ treatment needs and preferences facilitated by ROM, peer support and training of clinicians. The trial showed no effects of SDM-DI on Decisional Conflict, patient participation, working alliance and treatment adherence. However, compared to the control group, in the intervention group positive effects were reported regarding the application of SDM and symptom severity, which is the most important outcome of treatment. Furthermore, irrespective of the condition, this study demonstrated that when SDM was applied well, this approach led to less Decisional Conflict, which in turn had a positive influence on treatment outcome. Taken together, the study results pointed out that the application of a SDM intervention in the intake process targeting both patients’ and clinicians’ behaviour is a promising approach, which requires thorough implementation and continuation during treatment. Additionally, further exploration is needed to find out which interventions would help patients and clinicians best to foster patient participation in decision making about treatment.

## Registration

The trial is registered in the Dutch trial register with number: NTR5677 and registration date: 17 January 2016 (http://www.trialregister.nl/trialreg/admin/rctview.asp?TC=5677).

## Protocol

Full details of the trial protocol [16] can be found at: https://doi.org/10.1186/s12888-017-1247-9.

## Supporting information

S1 Protocol (PDF)

S1 CONSORT Checklist (DOCX)
